# Genetic studies of gestational duration and preterm birth

**DOI:** 10.1016/j.bpobgyn.2018.05.003

**Published:** 2018-10

**Authors:** Ge Zhang, Amit Srivastava, Jonas Bacelis, Julius Juodakis, Bo Jacobsson, Louis J. Muglia

**Affiliations:** a Division of Human Genetics, Cincinnati Children's Hospital Medical Center, USA; b The Center for Prevention of Preterm Birth, Perinatal Institute, Cincinnati Children's Hospital Medical Center, USA; c March of Dimes Prematurity Research Center Ohio Collaborative, USA; d Department of Pediatrics, University of Cincinnati College of Medicine, USA; e Department of Obstetrics and Gynecology, Sahlgrenska University Hospital Östra (East), Gothenburg, Sweden; f Department of Obstetrics and Gynecology, Institute of Clinical Sciences, Sahlgrenska Academy, University of Gothenburg, Gothenburg, Sweden; g Department of Genetics and Bioinformatics, Area of Health Data and Digitalisation, Norwegian Institute of Public Health, Oslo, Norway

**Keywords:** Gestational duration, Preterm birth, Complex human trait, Genome-wide association, GWA, genome-wide association, WES, whole-exome sequencing, WGS, whole-genome sequencing, SNP, single nucleotide polymorphism, MAF, minor allele frequency, LD, linkage disequilibrium, MR, Mendelian randomization, CRL, crown-rump length, LMP, last menstrual period, PROM, premature rupture of membranes, HLA, human leukocyte antigen, CNV, copy number variation, OD, obstetrical dilemma, EGG, energetics of gestation and growth

## Abstract

The fine control of birth timing is important to human survival and evolution. A key challenge in studying the mechanisms underlying the regulation of human birth timing is that human parturition is a unique to human event — animal models provide only limited information. The duration of gestation or the risk of preterm birth is a complex human trait under genetic control from both maternal and fetal genomes. Genomic discoveries through genome-wide association (GWA) studies would implicate relevant genes and pathways. Similar to other complex human traits, gestational duration is likely to be influenced by numerous genetic variants of small effect size. The detection of these small-effect genetic variants requires very large sample sizes. In addition, several practical and analytical challenges, in particular the involvement of both maternal and fetal genomes, further complicate the genetic studies of gestational duration and other pregnancy phenotypes. Despite these challenges, large-scale GWA studies have already identified several genomic loci associated with gestational duration or the risk of preterm birth. These genomic discoveries have revealed novel insights about the biology of human birth timing. Expanding genomic discoveries in larger datasets by more refined analytical approaches, together with the functional analysis of the identified genomic loci, will collectively elucidate the biological processes underlying the control of human birth timing.

Gestational duration is an important complex human trait controlled by multiple physiological processes. Despite years of research efforts, the biological mechanisms underlying human birth timing remain poorly understood. Genetic studies have the potential to provide new biological insights by identifying associated genes and pathways. In this review, we attempt to discuss several key topics related to the genetic research of human gestational duration.

## Why genetic study of gestational duration

### Genetic components of gestational duration

**Fig. 1:**
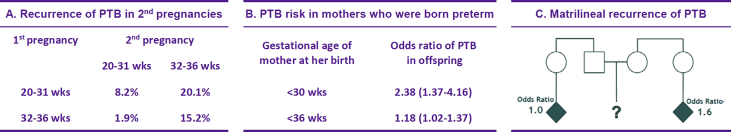
Recurrence risks of preterm birth (PTB) (Figure courtesy of Dr. Joe Leigh Simpson). **(A)** Women whose first deliveries were preterm have a higher chance of PTB in their second deliveries, especially when the first deliveries had shorter gestations [2]. **(B)** Mothers who themselves were born preterm have an increased risk of PTB [4]. **(C)** PTBs to a woman's mother, full sisters, or maternal half-sisters increase her PTB risk, whereas PTB in her paternal half-sisters or in members of her partner's family do not affect her risk [5].

Multiple lines of evidence have suggested a substantial genetic influence on the gestational duration and the risk of preterm birth [1]. Among the risk factors for a mother to deliver preterm, the most significant one is a history of previous preterm delivery [2]. Family studies also showed that women who were born spontaneously preterm or with siblings delivered in a similar manner have an increased risk of preterm delivery [3], [4], [5]. However, the risk is unaffected by the occurrence of preterm deliveries in woman's paternal half-sisters or in members of her partner's family, thus suggesting the substantial portion of the genetic risk passed through the female lineage [5]. The various recurrence risks are shown in Fig. 1. There is also a large residual risk of preterm birth in African American mothers after adjusting for sociodemographic factors, thus suggesting a potential contribution from biological factors such as genetics [6].

Twin studies [7], [8], [9] using birth data from different countries consistently demonstrated that approximately 30% of the variation in gestational duration or preterm birth is attributable to genetic factors. More comprehensive quantitative genetic studies using large birth registry data indicated that the majority of the genetic influence is due to maternal genes [10], [11]. Further, estimation of genetic and environmental contributions to gestational duration has been more extensively reviewed elsewhere [12].

### Biological insights – limitation of animal studies

Preterm birth, although empirically defined as a single clinical entity based on gestational age (<37 weeks), is the final outcome that can arise from many pathological processes with involvement of a wide variety of molecular pathways [13], ∗[14], [15]. The normal control mechanisms of parturition and how these mechanisms are disrupted by risk factors to cause prematurity remain poorly understood [16].

Up to now, our knowledge about the regulation of parturition mainly comes from animal studies [17]. A common theme learned from these animal studies is the essential role of progesterone: uterine quiescence is maintained by elevated progesterone and parturition is associated with a marked decline in circulating progesterone levels in many mammals. However, in human pregnancy, the maternal serum concentration of circulating progesterone does not fall with the onset of labor. Instead, there might be various molecular mechanisms that could constitute a functional withdrawal of progesterone [17], and it is unclear whether these changes are necessary and sufficient for human parturition [16].

The second characteristic of the commonly used animal models is the relatively abrupt transition from uterine quiescence to activation, probably triggered by a major hormonal switch (e.g., progesterone withdrawal). However, in human pregnancy, the transition from uterine quiescence to activation may be a more complex process controlled by many modular regulatory mechanisms [17].

These major differences make it difficult to extrapolate the regulatory mechanisms of parturition and the molecular etiologies of preterm birth from animal models to human. Indeed, different species have evolved different strategies to optimize their reproductive success, and many reproductive features have undergone substantial evolutionary divergence [18].

### Pregnancy and human evolution

The mechanics of human birth is also important in human evolution. Even compared with our close relatives, human birth has several distinctive features, including the orientation of the fetus as it passes through the birth canal, the way the fetus emerges from the birth canal, difficulty during labor, and the helpless state of the infants at birth [19]. It is widely believed that these characteristics are the consequences of the biological constraints imposed by human evolution: adaptation to bipedal locomotion decreased the size of the bony birth canal and the larger head became harder to fit through the narrow pelvis. This hypothesis, termed the “obstetrical dilemma” (OD) [20] posited that babies born earlier than the ideal is an evolutionary “trade-off” between bipedalism and larger brains. A study published in 2012 [21] proposed an alternative called the energetics of gestation and growth (EGG) hypothesis, which states that the duration of gestation is primarily determined by the balance between maternal energy supply and fetal energy demands and the timing of parturition is triggered by metabolic stress through hormonal signaling. Despite the differences, all these evolutionary hypotheses implicate strong natural selection acting on childbirth, in particular, related to the size of the baby and the duration of gestation.

Many physiological changes and disorders during or even after pregnancy can also influence the fitness of the mothers and infants. For example, gestational diabetes mellitus can cause macrosomia, in which the fetus grows too large to fit through the maternal pelvis [22]. Pre-eclampsia is the leading cause of maternal mortality and a major cause of preterm birth [23]. The prevalence of these disorders varies significantly in different populations, thus suggesting adaptation of metabolism during pregnancy to suit different environments worldwide [24].

### Gestational duration – defined by two genomes

**Fig. 2:**
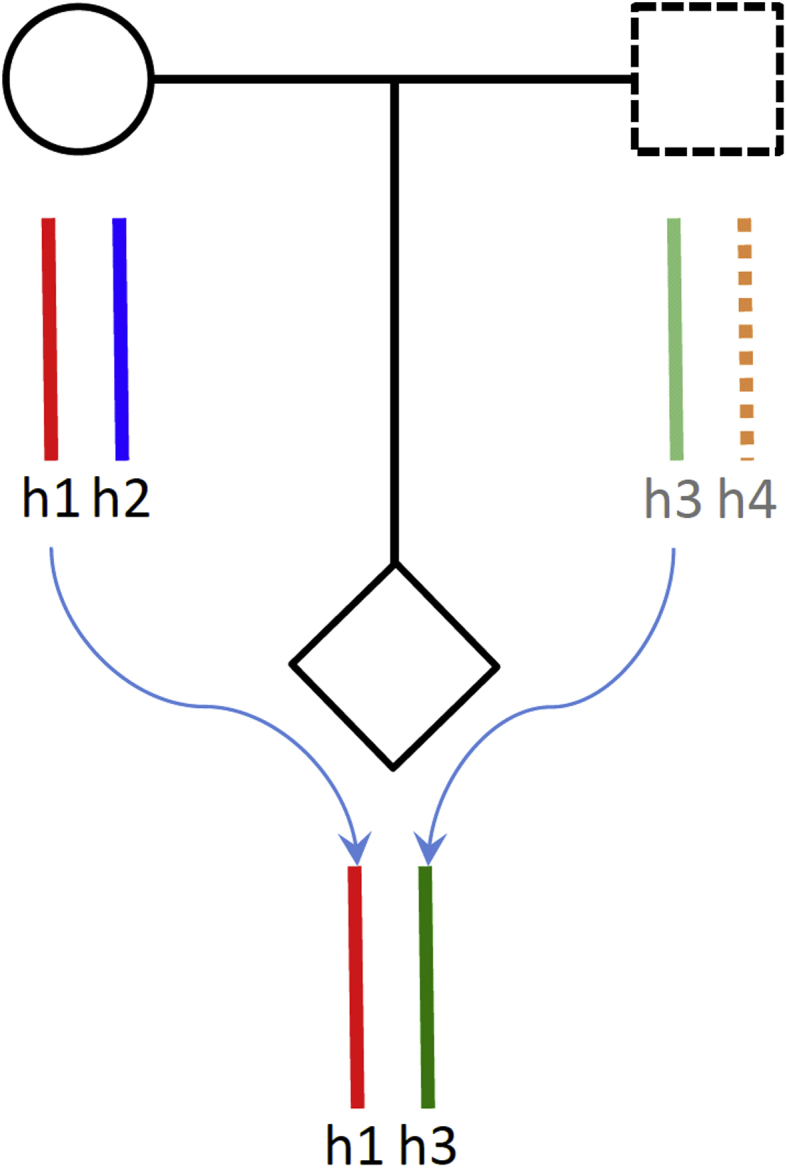
Haplotype transmission in a duo or trio.

Birth timing and pregnancy phenotypes are defined by both the maternal and the fetal genomes. These two genomes are correlated by sharing the maternally transmitted chromosomes (Fig. 2). The involvement of the maternal and fetal genomes and their different effects on pregnancy phenotypes has profound evolutionary implications. The most influential theory related to pregnancy might be the genetic conflicts hypothesis [25], which states that natural selection favors different outcomes for genes expressed in mothers and in fetuses. This theory has been used to explain many pregnancy complications and the evolution of genomic imprinting. Phenotype traits influenced by indirect genetic effects could also have distinct evolutionary trajectory [26]. In addition to these genetic effects, various molecular crosstalks take place between many different maternal and fetal tissues. These close interactions are important for successful pregnancy and parturition [27], [28]. As predicted by the co-adaptation model [29], selection on phenotypes with both maternal and fetal genetic influence can result in large genetic correlations and functional interactions between maternal and offspring characters.

## Genomic discoveries

Similar to other complex human traits, gestational duration is likely to be influenced by numerous genetic variants of small effect size and the detection of these small genetic effects requires very large sample sizes. In addition to the problems common for all the genetic studies of complex traits [30], several unique problems have made genetic studies of gestational duration and preterm birth even more challenging.

First, it is often difficult to accurately measure gestational age. Ultrasound measurement of fetal crown-rump length (CRL) in the first trimester is the most accurate method; however, this method still has a measurement error of ±5–7 days [31]. When ultrasound-based measure is not available, gestational age is determined by the date of the last menstrual period (LMP), which is more problematic because of inaccurate recall, irregularities in menstrual period, or variability in the timing of ovulation.

Second, parturition, term or preterm, could be initiated by various biological pathways and accompanied by different clinical presentations, e.g., with or without premature rupture of membranes (PROM) or other pregnancy complications. Despite their different clinical presentations, these subgroups may share similar biological mechanisms [32], which raised the question whether preterm birth should be split into more homogenous, albeit smaller, subgroups in genetic association studies.

Third, birth timing is influenced by maternal as well as fetal genome, which complicates the statistical genetic analysis. Several analytical approaches have been proposed, but none of these provide an optimal solution [33]. To date, most published studies tested genetic associations either in mothers or in infants separately. This strategy, however, does not integrate the maternal and fetal genetic effects.

Because of these difficulties, genetic studies of gestational duration and preterm birth have had limited success in the past decade. Only very recently did a large two-stage genome-wide study convincingly identify and replicate robust association of six genomic loci [34]. For other complex traits with similar health significance, the identified loci are far more than this number ∗[35], [36], [37], [38].

### Candidate gene studies

The initial effort to identify genetic associations with gestational duration or preterm birth was mostly based on a “candidate gene” approach, focusing on polymorphisms of a specific gene or a panel of genes with suspected functional relevance. A recent comprehensive review of 92 candidate gene studies published between 2007 and 2015 identified 119 candidate genes that have been suspected to be associated with preterm birth [39]. These genes were broadly grouped into six categories [1]: innate immunity and inflammation [2]; tissue remodeling and biogenesis [3]; endocrine system [4]; vascular and angiogenesis [5]; metabolism; and [6] miscellaneous. Similar to the conclusions of early systematic reviews [40], [41], [42], the authors found that although a large number of genes have been suspected, the results from the candidate gene studies are, by and large, inconsistent and inconclusive [39]. These candidate gene studies suffered from the common problems of the candidate gene approach [43] and did not conform to the reporting guidelines of genetic association studies [44]. Because of these concerns, investigators started to utilize a genome-wide approach to study the genetics of gestational duration, which presumably can provide an unbiased and more stringent solution [42].

### Genome-wide association (GWA) studies

The first published GWA study of spontaneous preterm birth utilized the phenotype and genotype data from the Danish National Birth Cohort [45]. Another study analyzed early spontaneous preterm delivery in a cohort with mixed genetic ancestry. The authors investigated maternal as well as fetal genomes and observed several significant associations; however, none of them were replicated in a validation cohort [46]. These initial failures mostly suggested that individual genetic variants associated with the duration of gestation only have small effects, and their detection would require much larger sample size [47].

To overcome sample-size limitations, an international academic collaboration with the direct-to-consumer genotyping company 23andMe conducted a two-stage GWA study [34]. In the discovery stage, they used data from more than 44,000 women who were research participants of 23andMe. They further replicated their discovery findings in ∼8000 samples collected from Denmark, Norway, and Finland. Through this study, they identified and replicated six genomic loci (*EBF1*, *EEFSEC*, *AGTR2*, *WNT4*, *ADCY5,* and *RAP2C*) in the maternal genome robustly associated with gestational duration and preterm birth (Table 1). They also confirmed the maternal origin of the observed genetic associations based on association tests using infant samples and joint maternal–fetal analysis.

**Table 1:** Six genetic loci associated with gestational length or preterm birth.

Chr	Locus	Functional relevance
5	*EBF1*	Regulates adipocyte differentiation and development; associated with birth weight
3	*EEFSEC*	Selenium metabolism; reduced selenium concentration is associated with preterm birth risk
1	*WNT4*	Important for the development of the female reproductive system; associated with endometriosis
X	*AGTR2*	Modulates uteroplacental circulation
3	*ADCY5*	Cell energy and metabolism
X	*RAP2C*	Associated with birth weight (in other studies)

Similar to many other GWA findings, the functional involvement of these identified loci has not been previously hypothesized. However, the functional roles of these loci in uterine development, maternal nutrition, and vascular control support their mechanistic involvement in birth timing control (Table 1), which highlighted the advantage of GWA in identifying novel genes and pathways. For example, the association at the *WNT4* loci suggests that cells within the lining of the uterus (i.e., endometrium) play an important role in the duration of pregnancy. Another identified gene region (*EEFSEC*) suggests that lack of selenium, rather than genes involved in selenoprotein synthesis, might affect preterm birth risk.

Like for most other human complex traits, the effect sizes of the six identified associations are uniformly small. In combination, these loci only explain <1% of phenotypic variation in duration of gestation or preterm birth risk [34]. Although these identified loci have no clinical diagnostic value in prediction of preterm birth, the major value of these GWA findings lays in the novel biological insights implicated by these identified gene loci.

More recently, by performing a large scale GWA meta-analysis of more than 80,000 infants from 19 studies, Liu et al. reported a locus on 2q13 in the fetal genome associated with gestational duration with genome-wide significance. The locus harbors genes encoding the proinflammatory cytokines IL-1α and IL-1β, which are central to the induction of uterine activation proteins that precede labor [48].

These large studies confirmed that GWA in large datasets is a feasible approach to identify genetic variants associated with gestational duration and preterm birth risk. It can be anticipated that, in foreseeable future, more genomic loci will be discovered and replicated by even larger GWA studies.

### Rare variant associations

During the past several years, advances in DNA sequencing technologies have enabled genome-wide assessment of rare genetic variants and their role in complex traits [49]. Several sequencing studies have investigated gestational duration, mostly on case-enriched pedigrees. The first study using whole-exome sequencing (WES) in spontaneous preterm birth was performed on 10 mothers from densely affected families [50]. The study found that the complement/coagulation factor pathway was enriched in harboring rare variants. Another WES study in more than 90 sister pairs found that 34 of the 95 (35.8%) families had one or more rare variants in the *HSPG2* gene shared by all sisters [51]. Huusko et al. [52] studied Finnish families and identified multiple *HSPA1L* rare variants in several families. Using high-coverage whole-genome sequencing (WGS) data from 816 parent–offspring trio families, Li et al. [53] found increased *de novo* mutation burden in preterm birth fetal genomes and those preterm-associated *de novo* mutations preferentially affect genes involved in early fetal brain development. As the cost of next-generation sequencing will continue to drop, WES or WGS studies in typical families or in large number of samples would eventually reveal many robust rare variant associations that would be missed by the GWA approach.

### Other genetic association studies

Epidemiologic studies in two European populations indicate that a substantial portion of preterm birth risk is maternally determined, thus suggesting a role of mitochondrial inheritance [5], [11]. An early case–control study in 400 Caucasian women indicated that a nonsynonymous variant in the mitochondrial genome may play a significant role in preterm birth by an interaction with smoking [54]. However, a later study with larger sample size failed to link preterm birth to specific polymorphisms in mitochondrial DNA [55]. More recently, Crawford et al. [56] examined whether the differences in the ancestral inheritance between mitochondrial and nuclear genome are associated with an increased risk of preterm birth, and they showed that infants with higher degrees of divergent ancestry were at increased risk of preterm birth. This finding may partially explain the increased risk of preterm birth in African American infants, and the mechanism may arise from the “mismatch” between haplogroup-defining mitochondrial polymorphisms with geographically local nuclear variation.

## Genomics as an epidemiological tool

**Fig. 3:**
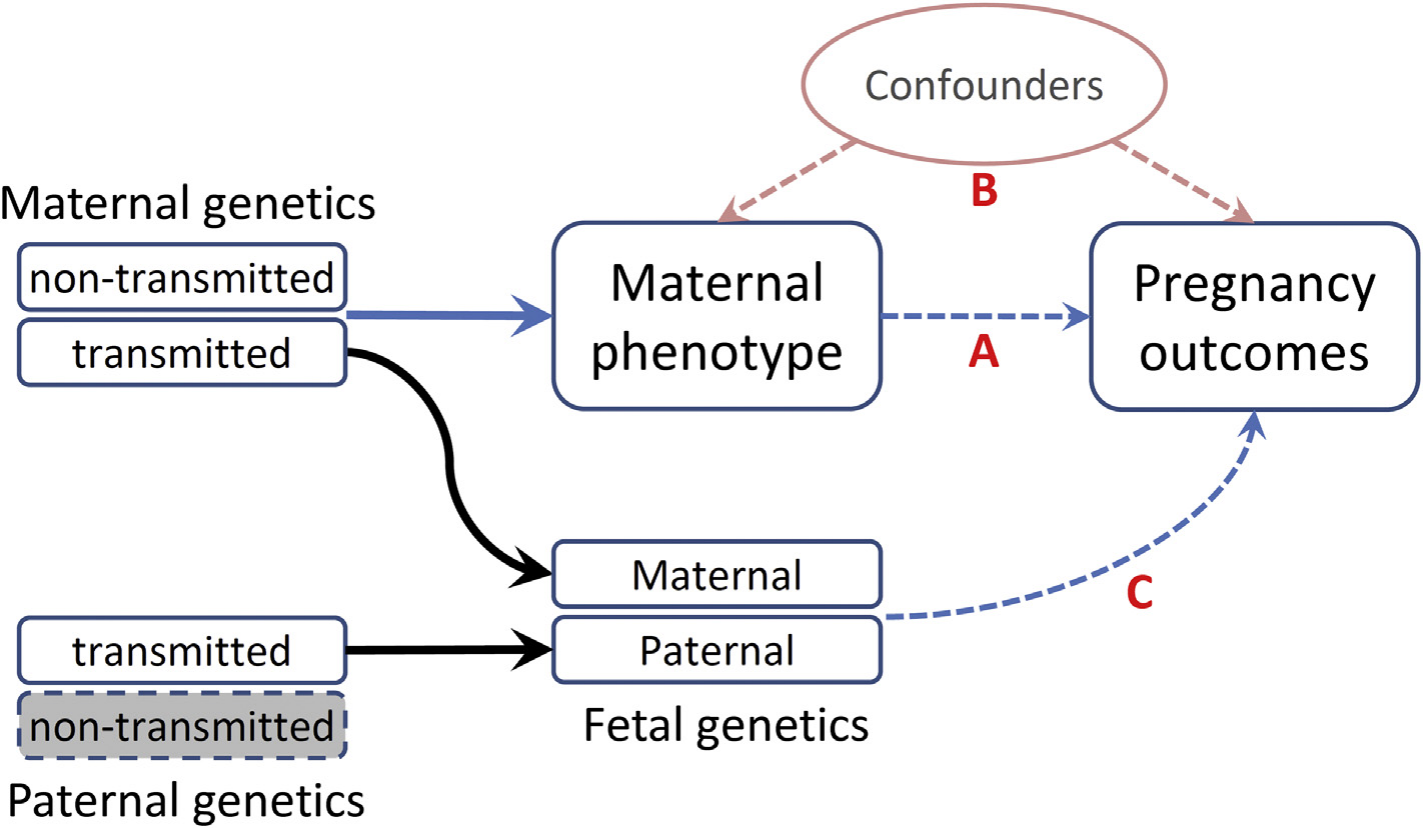
Possible causal mechanisms that can lead to the observational associations between a maternal phenotype and pregnancy outcomes. **(A)** Direct causal effect (maternal effect) of maternal phenotype on pregnancy outcomes. **(B)** Associations of social and nutritional confounders that have impacts on both maternal phenotype and pregnancy outcomes. **(C)** Fetal genetics that directly influences pregnancy outcomes.

A wide variety of maternal physical and physiological traits and environmental exposures have been associated with pregnancy outcomes. For example, maternal height, weight [57], blood glucose level [58], blood pressure [59], and exposure to various environmental hazards [60] have been reported to be associated with fetal growth and gestational duration. Different causal mechanisms have been proposed to explain the observed associations (Fig. 3); however, it is difficult to establish robust causal links by conventional epidemiological methods because of various confounding effects [61], ∗[62], ∗[63].

### Causal inference using Mendelian randomization

Genomic data offer the potential to disentangle causal relationships from epidemiological correlations using Mendelian randomization (MR) [64], [65]. This approach utilizes genetic variant(s) (i.e., genetic instrument) to interrogate the causal effect of the risk factor on an outcome. MR relies on three assumptions [1]: the genetic instrument is associated with the risk factor with enough strength [2]; the genetic instrument is not associated with confounders; and [3] the genetic instrument does not influence the outcome through other mechanisms (biological pleiotropy). MR has been successfully utilized in studying the relationships between many exposures/biomarkers and health outcomes [66].

### Using nontransmitted alleles

MR has also been used to study the impacts of intrauterine exposures on health outcomes in offspring [67], [68]. When utilizing MR to draw causal inference between maternal phenotype/exposure and outcomes in offspring, the approach is complicated by the transmission of maternal alleles (Fig. 3. Pathway C), and therefore, the fetal genotype needs to be adjusted for to avoid the confounding effects due to genetic sharing between mother and offspring ∗[62], [64], [69]. To overcome the problem, Zhang et al. [63] proposed a method to utilize the nontransmitted alleles as a valid genetic instrument, and thus to avoid the interference by genetic transmission in causal inference between maternal exposure and outcomes in offspring. Utilizing this method, they studied the causal relationship of maternal height with birth size and gestational duration. This approach is a novel extension of the MR methodology and can have many applications in casual inference between parental exposure and outcomes in offspring [70], [71].

## Integrated genomic analysis in mother/fetus pairs

Genetic studies usually aim to understand the relationship between genotype and phenotype in individual organisms. Accordingly, the default analytical or sampling units in these studies are individual organisms. Gestational duration and more broadly pregnancy phenotypes, however, are outcomes of a pregnancy event involving both mother and her fetus (sometimes more than one). This fundamental difference requires the analysis of pregnancy phenotypes using a different conceptual framework, in which mother/fetus duos (individual pregnancies) are more appropriate analytical units.

### Mother/fetus pairs as analytical units

**Fig. 4:**
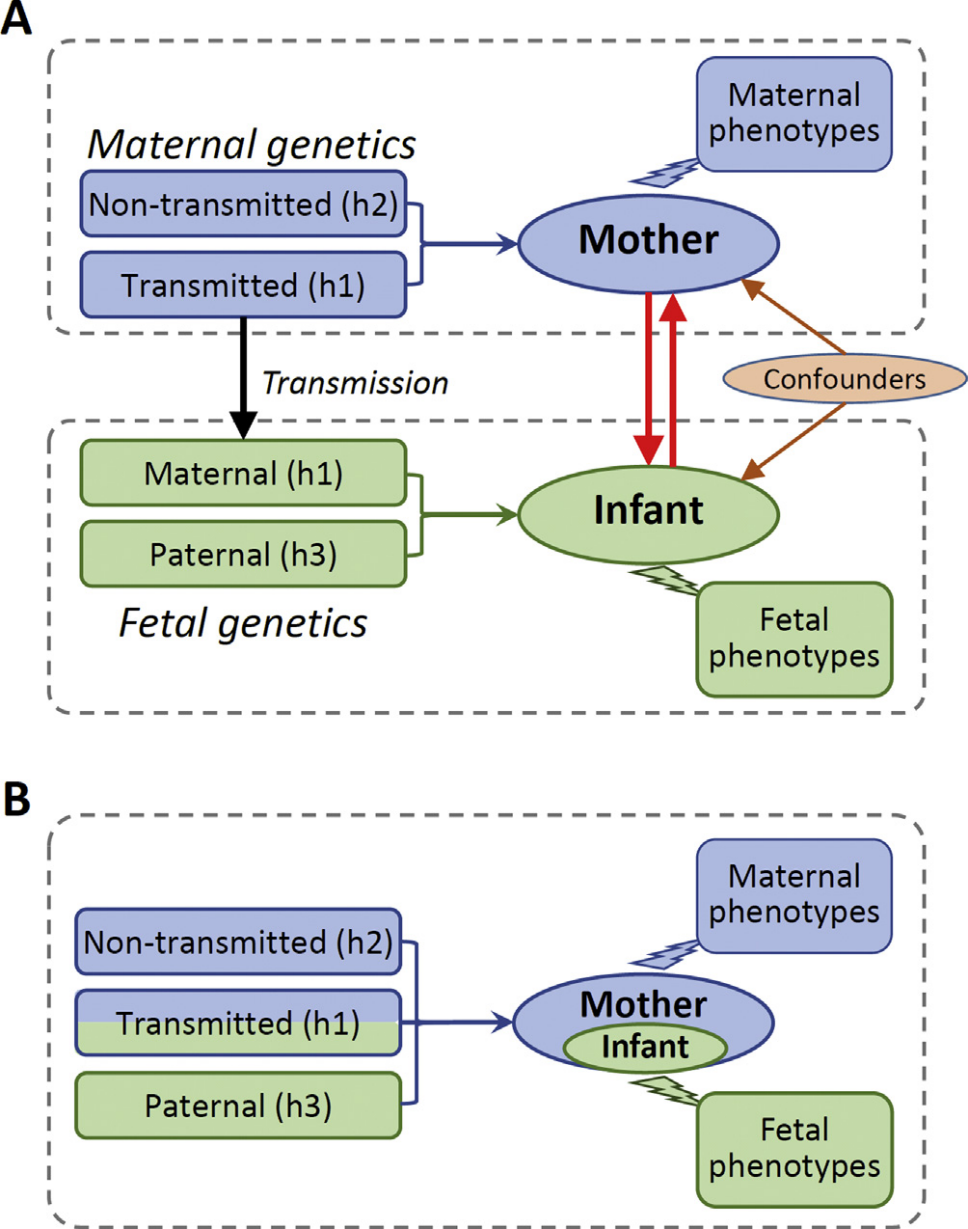
Conventional **(A)** and integrated maternal/fetal approach **(B)** to gestational phenotypes.

Conventional genetic methods usually regard maternal gestational phenotypes or fetal pregnancy outcomes exclusively pertaining either to mothers or infants and examine genetic associations separately in mothers or in infants (Fig. 4A). This conventional framework has several limitations [1]: it cannot model the genetic correlation between mothers and infants due to the sharing of maternally transmitted alleles [2]; it is less efficient in capturing the joint effects of maternal and fetal genome; and 3) it cannot reveal the crosstalk between mothers and fetuses.

Treating mother/fetus duo (pregnancy) as the analytical unit (Fig. 4B) provides a uniform solution to these problems. From a genetic perspective, in each such “unit,” there are three distinct haplotypes (alleles) (Figs. 2 and 4B). It should be noted that, the “haplotypes” here refer to the sets of transmitted or nontransmitted alleles. h1 and h3 correspond to the true haplotypes in the infants, but h1 and h2 are the recombinants in meiotic transmission and not the true haplotypes in the mothers. Each of these haplotypes can affect gestational phenotypes in different ways. h1 (maternal transmitted haplotype) can affect phenotype through mothers or/and fetuses; h2 (maternal nontransmitted haplotype) can only affect the phenotype through mothers and h3 (paternal transmitted haplotype) only through fetuses (if assuming no paternal effect). From a phenotypic perspective, all pregnancy phenotypes can be equally associated with the “unit” regardless their maternal or fetal origin. Under this analytical framework, we can conduct integrated genomic analysis of pregnancy phenotypes at three different levels.

### Genomic analysis at three different levels

#### Single-locus association test in mother/fetus duos

At the single-locus level, the joint effects of the three haplotypes (or alleles) on a pregnancy phenotype (*Y*) can be modeled by a linear model (*Y ∼ h1* + *h2* + *h3*). A list of questions related to the various genetic effects on a pregnancy outcome can be explicitly modeled and tested by different linear hypotheses of these three haplotypes. For example, the parent-of-origin effect in infants can be tested by a hypothesis H_0_: h1−h2 = h3 (which accounts for possible maternal effect of h1 and is more accurate than simply comparing h1 = h3). This method can be regarded as the genotype-based test in mother/fetus duos involving three alleles. The advantage of this method is the joint modeling of various maternal and fetal genetic effects.

#### Haplotype-based genetic score analysis

A complex trait is influenced by many genetic variants with small effects. One common method to analyze the cumulative effect of multiple variants is to construct a genetic score that could explain a considerable proportion of phenotypic variation. Usually, the genetic score of a trait of an individual is calculated as $S=\sum b_{i}G_{i},$where $G_{i}$ = 0, 1, or 2 is the genotype of the subject and $b_{i}$ is the estimated allelic effect. Extended to mother/infant pairs (Fig. 4B), three haplotype scores (based on h1, h2, and h3) can be constructed. As the proxies for complex traits also carry accurate parental origin information, these haplotype scores can help us to understand the causal relationships between pregnancy outcomes [63] and the development or genetic origin of late-onset disorders [72]. These haplotype scores also provide a powerful way to examine interactions between maternal and fetal genomes and interactions between genotype and environments.

#### Haplotype-based partitioning of genetic variance

To explain the “missing heritability” [73], [74], Yang et al. developed a method (genome-wide complex trait analysis (GCTA)) to estimate the variance of a complex trait explained by genome-wide single-nucleotide polymorphisms (SNPs) [75], [76]. By treating mother/fetus duos as analytical units, the GCTA method or the linkage disequilibrium (LD) score method can be extended to partition the genetic variance of gestational phenotypes to maternally transmitted alleles (h1), maternally nontransmitted alleles (h2), and paternally transmitted alleles (h3). This polygenic analysis, even though not able to implicate specific genetic variants or genes, will quantify the relative contributions of maternal and fetal genome and provide important information about the genetic architecture of pregnancy phenotypes and their correlation with other complex traits.

## Perspectives and conclusion

Genomic studies in large number of samples have revealed several genomic loci associated with gestational duration or risk of preterm birth. These genomic discoveries have already implicated novel insights into the biology of human birth timing. To enable more informative functional and pathway analysis, it is necessary to expand GWA discoveries in larger samples and to further develop the overall genetic architecture of human pregnancy maintenance and parturition.

### Sample size is the key

Similar to other complex human traits, sample size is the key to success of genomic study of human birth timing. It can be anticipated that the available samples, including mothers and infants, with both genomic data and gestational phenotype information will increase substantially in the next several years. These will include customers of direct-to-consumer genomic testing services who agree to contribute their data to research. Many companies currently offer noninvasive prenatal testing (NIPT), which will collect genomic data and pregnancy phenotype data from millions of mothers.

In academic settings, many birth cohorts (http://www.birthcohorts.net/) have been established to study the early-life developmental origins of diseases [77]. Compared with other study designs, cohort studies have major advantages in the genetic study of pregnancy outcomes. First, these studies usually collect biological samples from both mothers and fetuses, which enable the joint analysis of maternal and fetal genomes; second, these studies usually have more detailed phenotype and environmental exposure data related to pregnancy; and third, with long-term follow-up data, it is possible to explore the late health impacts.

In particular, lack of large samples is impeding our understanding of preterm birth genetics in non-Caucasian populations. Many African countries have high preterm birth rates [78]. In the US, African Americans also have higher preterm birth rate than other racial groups [79]. Normal-term gestational duration may be shorter in African and South Asian ancestry pregnancies than in Caucasian European women [80]. A complete understanding of the genetic basis behind the racial/ethnic disparity of preterm birth will require systemic sample collection and large-scale GWA studies in these underrepresented populations [81].

### Analytical considerations

#### Joint analysis of maternal and fetal genetic effects

The involvement of both maternal and fetal genomes presents a challenge in the genetic analysis of gestational duration and pregnancy outcomes. As discussed in Section 4, treating mother/fetus duos (pregnancies) as analytical units provides a conceptual framework for pregnancy phenotypes. Many statistical genetic methods can be extended under this framework to accommodate the integrated analysis of the maternal and fetal genome.

#### More complete coverage (rare variants, HLA, CNV etc.)

As indicated by many recent GWA studies complemented with WGS, variants associated with complex traits are overwhelmingly common [82]. Therefore, a major endeavor in the genomic study of gestational duration should be continuously searching for common-variant associations in large cohorts using SNP data. The new reference panels now enable imputation of variants with minor allele frequencies (MAFs) as low as 0.1% [83], [84], which offers the opportunity to identify rare variant associations.

Human leukocyte antigen (HLA) genes have been suspected to play an important role in pregnancy [85]. Because of technical difficulties, it is difficult to test association in HLA regions by SNP data [86]. In the past several years, efficient statistical methods have been developed to impute HLA alleles from SNP data [87]. These new analytical methods, together with the copy number variation (CNV) analysis [88] allow a more complete coverage of the genetic variation.

#### Time-to-event analysis

Gestational duration is, by its nature, a time-to-event variable. As such, a more appropriate statistical approach should be the time-to-event analysis (or survival analysis) [89]. As genetic variants might confer different effects at different gestational stages, stratified analysis by different gestational stages [e.g., extremely preterm (<28 weeks), very preterm (28 to <32 weeks), moderate preterm (32 to <37 weeks), term (38–42 weeks), and late term (>42 weeks)] might reveal different windows of sensitivity to genetic perturbations.

#### Stratified analysis to dissect heterogeneity

It is well known that male fetuses have on average shorter gestational duration and higher risk of preterm birth than female fetuses [90], [91], [92]. Mechanisms involving gender differences in fetal growth and sex-linked biochemical processes have been postulated to explain this difference. Different preterm birth subtypes, such as pPROM, might have distinct genetic causes [93]. More refined genetic association analysis in homogeneous subgroups stratified by gender, different clinical presentations (e.g., with or without PROM), or exposure to risk factors such as infection might contribute to the understanding of the heterogeneous mechanisms underlying birth timing control.

#### Integrated analysis of multiple pregnancy phenotypes

Many pregnancy outcomes such as birthweight and birth length are primarily defined by the duration of gestation. Gestational duration can also influence, or *vice versa* be influenced by, many maternal phenotypes and physiological changes during pregnancy. Therefore, integrated analysis of multiple pregnancy phenotypes could provide a more complete understanding about the shared genetic causes and interactions between these various phenotypes. Indeed, several gene loci associated with gestational duration have also been associated with birthweight [34].

### Functional studies: from genomic discoveries to biology

Genetic studies in human populations only identify genomic loci with naturally occurring variants statistically associated with a trait or a disease phenotype. These associated loci might implicate genes or pathways and guide biological insight. However, elucidation of the molecular, cellular, and physiological mechanisms behind a genetic association requires additional functional analysis. These “post-GWA” studies are usually highly domain specific and context dependent [94] and involve diverse bioinformatics and experimental strategies and techniques.

There are several challenges in the functional analysis of the genomic loci associated with gestational duration. First, the regulation of birth timing involves multiple maternal and fetal gestational tissues, and the gene expression patterns of these tissues were highly heterogeneous [95]. Large-scale, genome-wide functional genomic data of human gestational tissues are still very sparse to support informative functional interpretation. Another challenging fact is that, pregnancy is a period that has undergone dramatic physiological changes. During this period, the regulatory networks might be rewired to adapt to different stages of pregnancy. Ideally, the molecular functional studies should use tissue samples collected at different gestational stages. One ongoing difficulty is the challenge to obtain appropriate human gestational tissue samples before term. Differentiated induced pluripotent stem cells (iPSCs) provide an alternative approach; however, they may not reflect the in vivo physiological conditions and maternal–fetal communication.

Another challenge is related to our limited physiological knowledge about human birth timing. In order to bridge the gap between molecular phenotype (e.g., gene expression) and organismal phenotype (e.g., duration of gestation), it is necessary to investigate relevant cellular phenotypes or intermediate physiological phenotypes with more direct connections to specific biological processes. However, most of the physiology of pregnancy that we learned from animal models is not paralleled in human studies. Pathway or network analysis of many associated loci might provide clues about relevant biological processes and cell types.

## Conclusion

The significant health impact, the profound implications of birth timing in human evolution, and the genetic involvement of both maternal and fetal genomes make the genetics of human birth timing a medically significant, scientifically intriguing, and a challenging problem. Despite the many practical and analytical challenges, large-scale genomic studies will continue to reveal more associated genomic loci and the overall genetic architecture of human birth timing. In these studies, the integrated genomic analysis in mother/fetus pairs has unique strength to dissect various genetic effects. Functional analysis of the identified genomic loci, facilitated by the accumulation of gestational tissue- and stage-specific functional genomic data, together with the data generated from other omics studies will collectively provide a deeper insight into the key biological processes and cellular physiology of human birth timing.

**Practice points**

• There is a substantial genetic influence on the gestational duration and the risk of preterm birth.
• The biology underlying human birth timing control is complex, and animal studies only provide limited information.
• The duration of gestation is important to human health and also has many evolutionary implications.
• Genomic studies in large samples could reveal genomic associations and implicate novel biological insights.
• Genomics can be used as an epidemiological tool to study the causal relationship between maternal phenotypes (or exposures) and offspring outcomes.

**Research agenda**

• Sample size is the key to successful GWA studies, and replication in smaller but well-phenotyped samples is necessary to validate the findings.
• Duration of gestation is defined by maternal and fetal genomes — joint genomic analysis in mother/fetus pairs has unique strength in dissecting the various genetic effects.
• Integrated analysis of multiple pregnancy phenotypes will reveal the shared genetic causes and the interactions between these phenotypes.
• Functional genomic analysis aided by data generated from other omics studies will accelerate the translation from GWA findings to biological knowledge.

## Conflict of interest statement

All authors declare that they have no real, or potential, conflicts of interest.
